# The Role of Robot-Assisted Radical Prostatectomy in the Management of Prostate Cancer and Future Perspectives

**DOI:** 10.3390/cancers17193122

**Published:** 2025-09-25

**Authors:** Marco Rinaldi, Sebastiano Di Lena, Antonio Amodeo, Angelo Porreca, Alessandro Crestani

**Affiliations:** 1 Urology Unit, Santa Maria della Misericordia University Hospital, 33100 Udine, Italy; 2 Urology, Western Hospital Unit “San Pio”, 74011 Castellaneta, Italy; 3 Urology Unit, Veneto Institute of Oncology IOV-IRCCS, 31033 Castelfranco Veneto, Italy; antonio.amodeo@iov.veneto.it; 4 Department of Urology, Humanitas Gavezzani, 24125 Bergamo, Italy; info@angeloporreca.it; 5 Department of Medicine, Udine University, 33100 Udine, Italy

**Keywords:** robot-assisted radical prostatectomy, urinary incontinence, erectile function, nerve sparing, oncological outcomes

## Abstract

**Simple Summary:**

In this manuscript we discuss Robot-assisted radical Prostatectomy (RARP) as the treatment of choice for prostate cancer. We report how RARP has demonstrated functional benefits compared to open and laparoscopic surgical procedures, always maintaining at least an equality in terms of oncological safety. In particular we deal with some techniques that influence functional results, including nerve-sparing surgery, preservation of the lateral pelvic fascia, anterior and posterior reconstruction and preservation of the bladder neck. We highlight the advances of this procedure and the presence of some new robotic platforms that present some differences compared to the most widespread ones and which in some cases make robotic surgery more accessible even in centers with fewer resources and lower surgical volumes.

**Abstract:**

Robotic-assisted radical prostatectomy (RARP) has emerged as a leading surgical approach for localized prostate cancer in many centers worldwide. Leveraging minimally invasive techniques and advanced visualization, RARP has demonstrated benefits in perioperative and functional outcomes, and at least comparable, if not better, oncologic control relative to open radical prostatectomy (ORP) and laparoscopic radical prostatectomy (LRP). This review summarizes the current evidence on the efficacy, safety, and functional outcomes associated with RARP and discusses its role in contemporary prostate cancer management.

## 1. Introduction

Prostate cancer is one of the most common malignancies among men globally. For localized disease, radical prostatectomy remains a cornerstone of curative treatment alternative to radiotherapy.

The procedure consists of the removal of the prostate and seminal vesicles and the subsequent re-anastomosis of the bladder neck to the urethra. The removal of the obturator and iliac lymph nodes may also be performed simultaneously according to the classification of the risk of lymph node involvement which is evaluated with the use of specific nomograms [1].

Since the introduction of robotic systems, especially the da Vinci Surgical System, robot-assisted radical prostatectomy (RARP) has increasingly replaced ORP and laparoscopic approaches in clinical practice. The precision and ergonomic advantages of RARP offer potential benefits in perioperative recovery and functional preservation.

We conducted a selective literature review using PubMed, MEDLINE, and key journals.

## 2. Indications for Radical Prostatectomy

Radical prostatectomy is part of the active treatment management procedures for prostate cancer. It can be performed in patients with a life expectancy, based on comorbidities, greater than 10 years, and if the patient is not eligible for active surveillance (AS) protocols, or in cases where he does not accept them.

Radical prostatectomy is recommended when the disease is localized and presents at least an unfavorable intermediate risk according to the D’ Amico classification of biochemical recurrence risk (BCR) of localized and locally advanced disease updated by EAU 2025 (shown in Table 1). It may also be performed in cases in which the pathology is localized at favorable intermediate risk but presents a greater number of positive biopsy samples, or if the patient does not accept the greater risk of progression of the disease associated with active surveillance protocols [2]; see Table 1.

In case of high risk localized disease, radical prostatectomy is still an option even if it will present a higher risk of PSA failure and metastatic progression, but not all high-risk patients have a uniformly unfavorable prognosis [3]. So, in these cases, RARP should be offered as part of a conceivable multi-modal therapy.

Currently, the European guidelines do not give strong recommendations but still weakly recommend performing salvage radiotherapy associated with androgen deprivation therapy in cases where, after radical prostatectomy, the patient has a persistent PSA but has no evidence of metastasis [2].

## 3. Perioperative Advantages

Multiple studies have shown that RARP is associated with reduced blood loss, lower transfusion rates, shorter hospital stays, and decreased postoperative pain compared to ORP. A systematic review and meta-analysis by Ficarra et al. (2012) reported a mean blood loss reduction of approximately 400 mL with RARP and a significantly lower transfusion rate [4]. Additionally, RARP is associated with fewer wound complications due to its minimally invasive nature.

In addition, the conversion rates of RARP into open surgery have always been low, but they have also shown a trend in improvement over time, most likely due to an increase in surgical experience in this area, the progressive improvement of instrumentation, and the spread of robotic surgery [5].

## 4. Functional Outcomes

Recovery of urinary continence and sexual function are critical considerations.

High-definition 3D visualization and articulating instruments allow for refined dissection around the neurovascular bundles. Tewari et al. (2018) demonstrated that experienced surgeons performing RARP achieved continence rates exceeding 90% at 12 months and better early recovery of erectile function, particularly in younger patients undergoing bilateral nerve-sparing surgery [6].

A retrospective study based on data from twelve Dutch hospitals noted a lower incidence of erectile dysfunction in the RARP group compared to LRP (67.7% vs. 76.2%; *p* = 0.052) [7].

**Figure 1:**
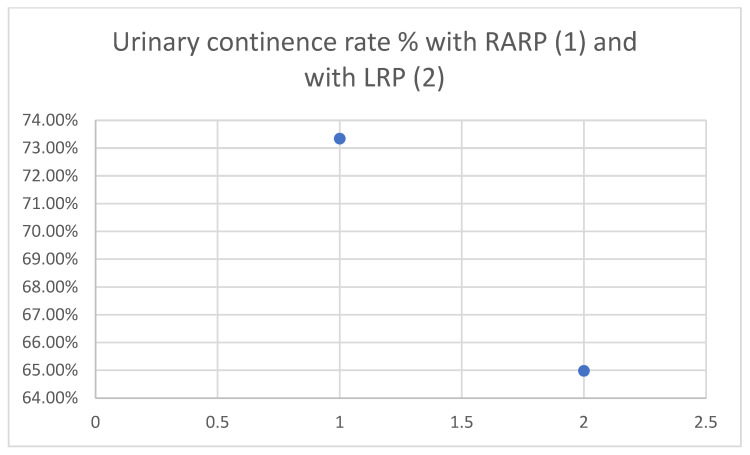
Urinary continence rate % with RARP (1) and with LRP (2). Data from the systematic review and meta-analysis by Lan Cao et al. (2019) [8].

RARP is associated with improved urinary continence outcomes. As shown in Figure 1, a national retrospective study reported better urinary function scores for RARP compared to LRP (mean EPIC score: 73.34 vs. 64.98; *p* = 0.002), with a higher proportion of patients achieving continence [8].

## 5. Nerve-Sparing RARP

Nerve-sparing (NS) RARP aims to preserve erectile function and urinary continence without compromising oncological control. Since the original description by Walsh and Donker in the early 1980s, NS techniques have evolved significantly with the advent of minimally invasive approaches, in particular RARP [9].

The bilateral neurovascular bundles (NVBs), running posterolaterally to the prostate, are critical structures that must be meticulously preserved during surgery to maintain postoperative potency.

A careful preoperative assessment is essential for identifying candidates for NS RARP. Key considerations include preoperative erectile function (for example, using the International Index of Erectile Function), tumor characteristics (clinical stage, PSA, biopsy Gleason grade group), prostatic MRI (particularly concerning extraprostatic extension), and laterality of disease.

The robotic approach of NS has facilitated enhanced visualization and precision, supporting more refined NS techniques.

**Figure 2:**
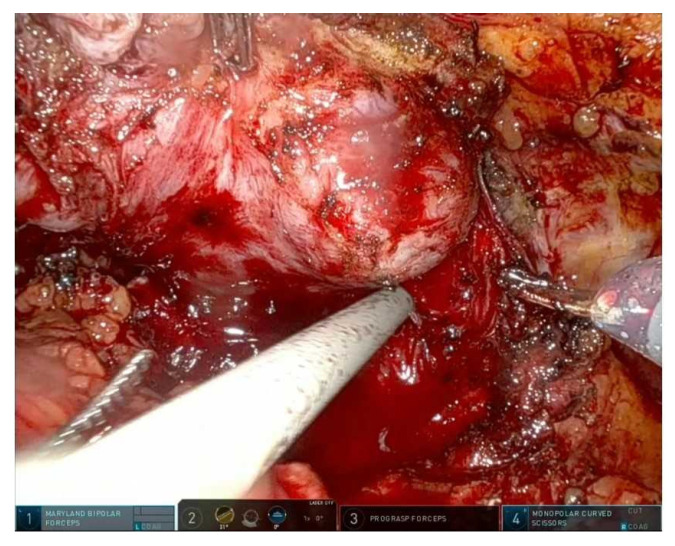
Clipless right prostate pedicle section and develop of right intrafascial plane.

**Figure 3:**
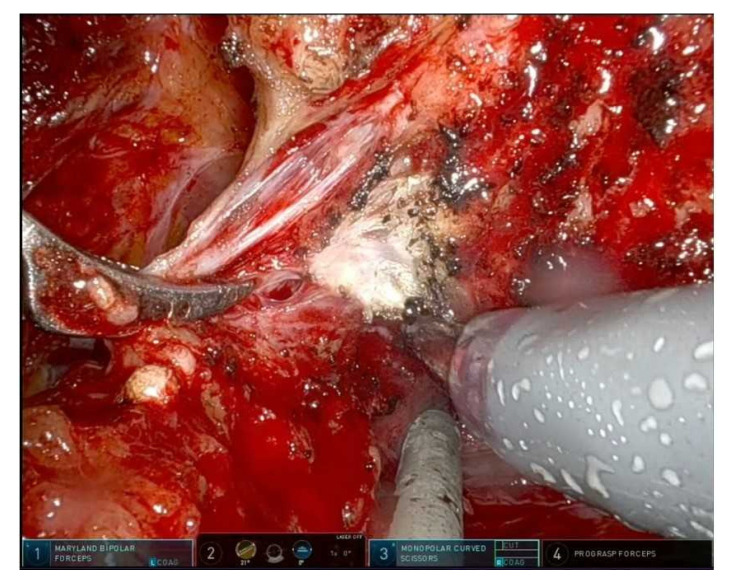
Clipless left prostate pedicle section and beginning of left interfascial plane.

There are three main planes of NVB dissection:

1. Intrafascial: maximal preservation, suitable for low-risk disease with favorable anatomy; Figure 2 shows an intrafascial dissection phase;
2. Interfascial: partial preservation, balances functional and oncological outcomes; Figure 3 shows a frame during interfascial dissection;
3. Extrafascial: non-NS dissection, reserved for high risk or extracapsular disease [10]. Figure 2 and Figure 3.

High anterior release, Veil of Aphrodite technique, and incremental nerve-sparing grading systems (Tewari’s grades 1–4) are examples of refined techniques that allow customization based on tumor risk and anatomic variations [11].

Oncologic safety is maintained in appropriately selected patients. Studies have shown that NS does not increase the risk of positive surgical margins when careful perioperative and intraoperative judgment is applied [12].

## 6. Lateral Pelvic Fascia Preservation Technique

Growing anatomical insight into the multilayered lateral pelvic fascia (LPF)—rich in neurovascular and smooth muscle fibers—has led to targeted preservation techniques postulated to support continence and potency through retention of periurethral innervation [13].

The lateral pelvic fascia comprises a fibrovascular multilayer covering the antero-lateral prostate capsule, harboring nerve fibers contributing to the membranous urethral sphincter and autonomic support structures [13]. In practice, the technique involves intrafascial or interfascial dissection to leave the LPF intact (Figure 2 and Figure 3). A specific approach, the modified apical dissection with lateral fascia preservation (mod-RARP), avoids aggressive apical dissection and maintains periurethral tissue around the urethral stump, preserving the NVB and lateral fascia envelope [14].

A retrospective comparison of lateral pelvic fascia preservation (LPFP) versus standard RARP (68 vs. 71 patients) demonstrated significantly faster continence recovery, with shorter time to continence, reduced operative time, and lower intraoperative blood loss; oncologic outcomes were similar between groups [15].

A separate study of endopelvic fascia preservation vs. standard RARP in 138 patients reported 97.1% continence at 12 months in the preservation group vs. 88.4% in controls (*p* < 0.05), and preservation was the only independent predictor in multivariate analysis [16].

Another single-surgeon series comparing fascia-sparing PFS-RARP (n = 239) to standard RARP (n = 102) found time to full continence reduced from 261 to 91 days and a 66% reduced risk of incontinence (0–1 pad), with no significant difference in biochemical recurrence [17].

Regarding sexual function, a feasibility series (35 potent men) undergoing prostatic fascia preservation reported that 97% retained erections sufficient for vaginal penetration at 12 months [13].

Unlike previous evidence, a randomized controlled trial of 158 men at Tampere University Hospital, comparing endopelvic fascia preservation vs. standard RARP, reported no significant difference in urinary continence or sexual function at 12 months [18].

So, applying intrafascial or interfascial that preserve lateral pelvic fascia and periurethral tissues can accelerate urinary continence recovery. Modified apical techniques further spare NVB and supporting fascia. Outcomes appear better in high-volume centers and in experienced hands, particularly for more refined LPF preservation techniques.

## 7. Posterior Reconstruction and Anterior Suspension Techniques

Posterior reconstruction involves approximating Denonivilliers’ fascia to the posterior aspect of the rhabdosphincter and/or anchoring to the bladder neck. This cranially repositioning of the urethral sphincter allows to improve pelvic support and reduces tension to the vesico-urethral anastomosis [19].

Several studies report different surgical techniques for posterior reconstruction, with the aim of improving continence recovery.

Nguyen et al. reported continence at 3 days (34% vs. 3%) and 6 weeks (56% vs. 17%), favoring posterior reconstruction, with average restoration of membranous urethral length by 2 mm [20].

By 3 and 12 months, differences tend to diminish, with many studies showing no long-term benefit compared to standard technique [19].

Anterior suspension, following ligation of the dorsal venous complex, secures the rhabdosphincter to peri-pubic structures (pubic bone, cooper’s ligament) providing an anterior hammock support [19].

Anterior suspension according to Patel demonstrated improved continence at 3 months (92.8% vs. 83%, *p* = 0.013) vs. controls [19].

This is a combination that reconstructs Denonvilliers’ fascia and the posterior bladder wall first, performs tension-free anastomosis, then suspends the bladder neck to arcus tendineus/puboprostatic plate with continuous suturing. This respects anatomical alignment and enhances both anterior and posterior support [21].

Systematic reviews claim that total reconstruction shortens time to continence and increases early continence rates more than posterior or anterior technique alone, with comparable complication or positive-margin rates [22].

A large volume but monocentric study reports a statistically significant better return to urinary continence, even in the long term (up to 48 months), in patients undergoing RARP with both anterior and posterior reconstructions compared to those undergoing RARP without any type of reconstruction [23].

## 8. Bladder Neck Preservation Technique

The internal sphincter at the bladder neck plays a critical role in passive urinary continence. Bladder neck preservation (BNP) in RARP aims to retain the internal urethral sphincter and urothelial coaptation zone, potentially accelerating urinary continence recovery.

In a prospective, randomized, single-blind trial involving 208 men, full bladder neck preservation let to significantly lower urine loss at 3, 6, and 12 months, higher social continence rates (84.2% vs. 55.3% at 3 months, rising to 94.7% vs. 81.4% at 12 months; all *p* < 0.05), and better quality of life scores. There was no significant difference in positive surgical margin rates between groups (12.5% vs. 14.7%, *p* = 0.65) [24].

A single-surgeon cohort of 233 RARP patients with bladder neck sparing showed a median blood loss of 75 mL, early catheter removal, and at six weeks, 69% were pad-free with quality of life improving significantly; surgical margin negativity stood at 85% [25].

Meta-analytic data indicate that BNP accelerates continence recovery, especially within the first 3–6 months, without increasing margin positivity. While long-term (12 months) continence rates eventually converge, early benefit is clear [26].

European association of urology (EAU) guidelines suggest that bladder neck preservation should be performed routinely when the cancer is distant from the base. However, bladder neck preservation cannot be performed in the presence of a large median lobe or a previous transurethral surgery procedure of the prostate [2].

## 9. Oncological Efficacy

While RARP offers clear perioperative advantages, oncologic outcomes are paramount. Several large cohort studies, including data from the SEER-Medicare database, have shown that RARP achieves similar positive surgical margin (PSM) rates and long-term biochemical recurrence-free survival compared to ORP. A prospective by Yaxley et al. (2016) found no significant difference in oncologic outcomes between RARP and ORP at 24 months [27].

Notably, in patients with higher-stage disease (pT3/4), RARP has been associated with a lower risk of PSA recurrence [8].

A recent network meta-analysis encompassing 80 studies found that RARP had a significantly lower PSM rate compared to ORP (relative risks (RR) 0.893, 95% credible intervals (Crl) 0.807–0.985).

The same meta-analysis reported lower BCR rate for RARP compared to ORP (RR 0.713, 95% Crl 0.587–0.869) and LRP (RR 0.672, 95% Crl 0.505–0.895), indicating improved oncological control with RARP [28].

## 10. Limitations and Learning Curve

Despite its advantages, RARP is not without limitations. High capital and maintenance costs may restrict its availability, particularly in low-resource settings. Additionally, outcomes are highly operator-dependent. Proficiency in RARP requires a steep learning curve, often estimated at 150–250 cases for consistent oncologic and functional outcomes [29]. Moreover, the learning curve for RARP depends on surgical volume at both individual and institutional levels. High-volume institutions demonstrate significantly improved perioperative metrics (short operative time, estimated blood loss) and more favorable oncological outcomes [30]. Low-volume centers often require more cases to reach comparable results; structured mentorship and previous laparoscopic experience can mitigate the disparity [31].

## 11. Future Directions

Technological advancements, including image-guided surgery, augmented reality, and real-time intraoperative margin assessment, continue to evolve. Additionally, several manufacturers have developed new robotic platforms, and their diffusion may reduce costs and increase accessibility, but there is still a need for time and studies that demonstrate the comparable effectiveness of these new robotic platforms with the da Vinci Surgical system.

Between the various platforms that are gaining ground are Hugo RAS (by Medtronic) and Versius (by CMR).

Hugo RAS is a modular system that offers the ability to operate using up to four bogies per arm, an approach that can offer greater flexibility and adaptability.

Also, Versius is a modular system and features an open console that allows the surgeon to adopt an ergonomic position both while standing and sitting for the entire duration of the operation; this allows the operator to decide how he wants to work and helps reduce the physical effort required to perform an operation.

The meta-analysis of Chen SY et al. (2025) aggregates perioperative and early postoperative data, including four studies totaling 145 patients undergoing Versius-assisted RARP, and shows perioperative safety and oncologic validity comparable to conventional RARP benchmarks (e.g., those derived from da Vinci-based case series), at least in low-volume, early-stage cohorts [32]. It showed a continence recovery by 3 months that appears favorable, aligning with current multiportal robotic surgery standards, and a positive surgical margins rate within expected ranges for RARP.

In addition, the study of Gavi F. et al. (2025) concluded that the Hugo RAS robotic system allows a successful and safe RARP, and that prior experience with robotic surgery, specifically the da Vinci system, positively influences perioperative outcomes in robotic-assisted radical prostatectomy [33]. Indeed, surgeons with more extensive experience exhibited shorter operative times, reduced blood loss, a quicker recovery, and fewer complications.

Therefore, there is already evidence, but there is a need for further and extensive studies that allow these new platforms to be compared with the most widespread (da Vinci surgical system).

Another emerging minimally invasive surgical technique is Single-port robot-assisted radical prostatectomy (SP-RARP), using a single access incision. This da Vinci system obtained the Food and Drug Administration (FDA) approval in 2018 and is available in Europe from 2024 [34]. Early clinical experiences suggest that SP-RARP is feasible and safe, with perioperative outcomes comparable to traditional multi-port approaches, while potentially offering benefits in reduced pain, shorter hospital stays, and improved cosmesis [35]. However, limitations include a learning curve impacting initial operative time and margins, limited long-term data, and equipment cost [36]. However, widespread adoption awaits more robust long-term data, cost-effectiveness assessments, and broader surgical proficiency. Technological enhancements and collaborative training programs will be key to optimizing outcomes.

## 12. Conclusions

RARP represents a safe and effective treatment for localized prostate cancer, offering excellent perioperative benefits, and at least an equivalent oncologic control to traditional approaches. These overall benefits position RARP as a favorable option for patients undergoing radical prostatectomy for localized prostate cancer. With ongoing innovations and surgeon training, RARP will likely continue to play a central role in the surgical management of prostate cancer.

## Figures and Tables

**Table 1:** D’ Amico classification of biochemical recurrence risk (BCR) of localized and locally advanced prostate cancer updated by EAU 2025 [2].

Low-Risk	Intermediate-Risk		High-Risk	
	Favourable	Unfavourable		
ISUP grade 1 and PSA < 10 ng/mL and cT1-2a	ISUP grade 2 and PSA < 10 ng/mL and cT1b-2b Or ISUP grade 1 and PSA 10–20 ng/ml and cT1b-2b Or ISUP grade 1 and PSA < 10 ng/mL and cT2b	ISUP grade 2 and PSA 10–20 ng/mL and cT1b-2b Or ISUP grade 3 and cT1-2b	ISUP grade 4/5 Or PSA > 20 ng/mL Or cT2c	cT3-4 and/or cN+ (any ISUP grade any PSA)
Localised				Locally advanced
